# AI-Driven Colon Cleansing Evaluation in Capsule Endoscopy: A Deep Learning Approach

**DOI:** 10.3390/diagnostics13233494

**Published:** 2023-11-21

**Authors:** Miguel José Mascarenhas Saraiva, João Afonso, Tiago Ribeiro, Pedro Cardoso, Francisco Mendes, Miguel Martins, Ana Patrícia Andrade, Hélder Cardoso, Miguel Mascarenhas Saraiva, João Ferreira, Guilherme Macedo

**Affiliations:** 1 Department of Gastroenterology, São João University Hospital, 4200-427 Porto, Portugal; joaoafonso28@gmail.com (J.A.); tiagofcribeiro@outlook.com (T.R.); pedromarilio@gmail.com (P.C.); francisco.cnm@gmail.com (F.M.); miguel.pedro96@gmail.com (M.M.); anapatriciarandrade@gmail.com (A.P.A.); hc@sapo.pt (H.C.); guilhermemacedo59@gmail.com (G.M.); 2 Gastroenterology and Hepatology, WGO Gastroenterology and Hepatology Training Centre, 4050-345 Porto, Portugal; 3 Faculty of Medicine, University of Porto, 4169-007 Porto, Portugal; 4 Endoscopy and Digestive Motility Laboratory, ManopH, 4000-432 Porto, Portugal; miguelms.manoph@gmail.com; 5 Department of Mechanical Engineering, Faculty of Engineering, University of Porto, 4169-007 Porto, Portugal; j.ferreira@fe.up.pt; 6 INEGI—Institute of Science and Innovation in Mechanical and Industrial Engineering, 4200-465 Porto, Portugal; 7 DigestAID—Digestive Artificial Intelligence Development, 4200-135 Porto, Portugal

**Keywords:** capsule endoscopy, colon capsule endoscopy, artificial intelligence, bowel preparation, deep learning

## Abstract

Gastroenterology is increasingly moving towards minimally invasive diagnostic modalities. The diagnostic exploration of the colon via capsule endoscopy, both in specific protocols for colon capsule endoscopy and during panendoscopic evaluations, is increasingly regarded as an appropriate first-line diagnostic approach. Adequate colonic preparation is essential for conclusive examinations as, contrary to a conventional colonoscopy, the capsule moves passively in the colon and does not have the capacity to clean debris. Several scales have been developed for the classification of bowel preparation for colon capsule endoscopy. Nevertheless, their applications are limited by suboptimal interobserver agreement. Our group developed a deep learning algorithm for the automatic classification of colonic bowel preparation, according to an easily applicable classification. Our neural network achieved high performance levels, with a sensitivity of 91%, a specificity of 97% and an overall accuracy of 95%. The algorithm achieved a good discriminating capacity, with areas under the curve ranging between 0.92 and 0.97. The development of these algorithms is essential for the widespread adoption of capsule endoscopy for the exploration of the colon, as well as for the adoption of minimally invasive panendoscopy.

## 1. Introduction

Diseases of the colon, especially colorectal cancer (CRC), are major health issues worldwide, particularly in developed countries. A colonoscopy is the gold standard to study anomalies in the colon, despite being an invasive and, at times, painful procedure (potentially requiring sedation) associated with a risk of bleeding or perforation [1]. However, Colon Capsule Endoscopy (CE), in conjunction with technical advances, is steadily becoming a minimally invasive alternative to evaluate the colon mucosa, with the potential to overcome these drawbacks [2,3]. Indeed, the recent advances in CE have shown that this procedure can replace conventional colonoscopy, yielding better results than other approaches like computed tomography colonography (virtual colonoscopy) without the radiation hazard [4].

CE examinations can produce large numbers of images (over 50,000), making them time-consuming (approximately 50 min per exam) and laborious to review [3]. Moreover, any given frame may only capture a fragment of any mucosal abnormality, and lesions may only be evident in a few frames, reflecting the risk of overlooking important lesions [3]. The value and reliability of a CE is also dependent on the colon’s cleanliness, achieved in the preparation for the procedure, which may compromise the reliability of the examination if inadequate. This is perhaps more critical in CE than in colonoscopy, as the capsule does not have the ability to insufflate the lumen and clean debris. Thus, it is essential to be able to assess the quality of colon preparation reliably, objectively and reproducibly in CCE to avoid the need of having to revert to diagnostic colonoscopy.

In recent years, notable advances have been made in applying automated AI algorithms to aid in the reading of CE examinations, principally focusing on disease identification and classification [5,6]. Multilayered algorithms such as convolutional neural networks (CNNs) are designed for automatic image analysis and, as such, there has been much interest in using automated CNN-based tools to examine CE videos. Indeed, CNN-based algorithms appear to show good diagnostic yields for the real-time detection of colorectal neoplasia in conventional colonoscopy [7] and to identify polyps in CCE [7,8,9], with good accuracy, sensitivity and specificity. Similarly, the detection of blood or hematic residues in the lumen of the colon is important when reviewing CE images. However, to date, there have been few attempts to develop tools to automatically assess cleanliness in CE examinations [10,11]. Thus, we set out to design and develop a pioneering CNN-based algorithm to provide an automatic categorization of the quality of colon preparation using CCE images and to validate the performance of this tool on a large set of real-world CCE images.

## 2. Materials and Methods

### 2.1. Study Design

Colonic images from capsule endoscopy (CCE) were obtained from patients examined between 2015 and 2022 at Centro Hospitalar Universitário de São João (CHUSJ, Porto, Portugal) and the ManopH Gastroenterology Clinic (MGC, Porto, Portugal), and they were reviewed retrospectively. The complete videos of 141 examinations were reviewed. A total of 35,269 frames of the colon mucosa were retrieved. This study received approval from the Ethics Committee of the São João University Hospital/Faculty of Medicine of the University of Porto (Approval No. CE 407/2020) and adhered to the principles outlined in the Helsinki Declaration. This retrospective non-interventional study involved the removal of any potentially identifying information related to the subjects. Each participant was assigned a randomly generated code to safeguard data anonymity, ensuring compliance with the General Data Protection Regulations of the European Union. Furthermore, data confidentiality was confirmed by experts holding a Data Protection Officer certification from Maastricht University, making the data non-traceable. 

### 2.2. Capsule Endoscopy and Colon Capsule Endoscopy Procedures

Capsule endoscopy (CE) procedures were performed with one of four distinct CE systems, namely PillCam SB3™ (Medtronic, Minneapolis, MN, USA) and OMOM HD™ (Jinshan Science & Technology Co., Chonqing, China), as well as two systems designed for CCE: PillCam COLON2™ (Medtronic, Minneapolis, MN, USA) and PillCam Crohn’s Capsule™ (Medtronic, Minneapolis, MN, USA). Images were reviewed using the PillCam™ software version 9.0 (Medtronic, Minneapolis, MN, USA) and the Vue Smart Software (Jinshan Science & Technology Co., Chonqing, China). Every acquired image was processed to safeguard against potential identification, including sensitive details such as name, ID number, and the procedure date. Furthermore, each extracted frame was sequentially numbered prior to being securely stored. Patients undergoing CCE examination underwent bowel preparation in compliance with the European Society of Gastrointestinal Endoscopy (ESGE) guidelines (Rondonotti et al. 2018 [12]). Summarily, patients followed a clear liquid diet over the two days prior to their procedure, with overnight fasting before the examination. Each patient underwent preparation with a split-dose regimen of a sodium picosulfate combination (sodium picosulfate, magnesium oxide and citric acid; Citrafleet^®^, Jaba Recordati, Porto Salvo, Portugal). Patients undergoing conventional CE (Pillcam SB3™, Minneapolis, USA and OMOM HD™, Chongqing, China) received a single dose of the same preparation immediately before the procedure. Water with simethicone was given to prevent foaming. After ingestion of the capsule, the patients were not allowed to eat for at least 4 h. If the capsule remained in the stomach 1 h after ingestion, prokinetic therapy was applied (10 mg domperidone, up to 30 mg), in line with ESGE recommendations [12].

### 2.3. Classification of Bowel Preparation

**Figure 1:**
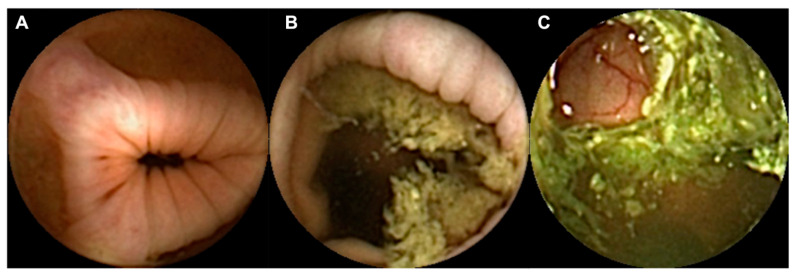
Images depicting the quality of bowel preparation. (**A**)—excellent; (**B**)—satisfactory; (**C**)—unsatisfactory.

Three experienced gastroenterologists (M.M.S., H.C. and A.P.A.), specialized in capsule endoscopy (CE), each having previously reviewed more than 1500 CE exams, independently assessed still frames. They evaluated the colon cleansing quality by considering the proportion of visible mucosa and the extent of obscuration via factors such as bubbles, bile and debris. The colon preparation quality in each still frame was classified as follows: it was excellent when ≥90% of the mucosa was visible, satisfactory when 50 to 90% of the mucosa was visible, and unsatisfactory when <50% of the mucosa was visible (refer to Figure 1 for visual representation). To establish an agreement, two of the three experts needed to concur on the assessment for each frame.

### 2.4. Development of the Convolutional Neural Network

The CNN was created by building upon the *Resnet18* model and then trained using the ImageNet dataset. We retained the model’s convolutional layers to leverage the knowledge gained from ImageNet. However, we removed the final fully connected layers and replaced them with new fully connected layers, matching the number of classes used to classify the CCE images. We incorporated two blocks, each featuring a fully connected layer, followed by a dropout layer with a 0.3 dropout rate. Following these two blocks, we added a dense layer, defining its size to match the number of classification categories (three in this case).

The learning rate was determined using the LRFinder algorithm [13]. We employed a batch size of 64 and conducted 15 training epochs. Data preparation and model execution were facilitated through the PyTorch Lightning library.

These computations were performed on a computer equipped with a 2.1 GHz Intel Xeon Gold 6130 processor (Intel, Santa Clara, CA, USA) and a dual NVIDIA RTX A6000 48 GB graphics card (NVIDIA Corporate, Santa Clara, CA, USA).

### 2.5. Model Performance and Statistical Analysis

**Figure 2:**
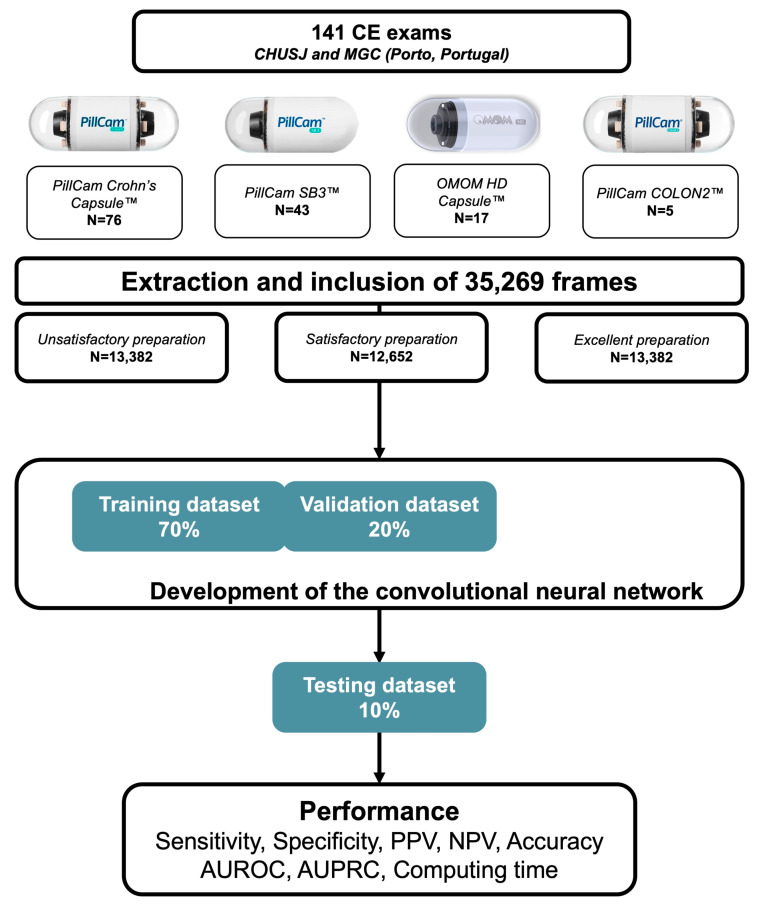
Study design indicating the proportion of the examinations carried out with each type of capsule. The level of cleanliness was classified as follows: excellent (≥90% of the mucosa visualized); satisfactory (50–90% of the mucosa visualized); and unsatisfactory (<50% of the mucosa visualized). Abbreviations: CE, capsule endoscopy; PPV, positive predictive value; NPV, negative predictive value; AUC; area under the ROC curve.

The images collected (*n* = 35,269) were split into training, validation and independent testing datasets, according to ratios of 70%, 20% and 10%, respectively, following a patient-split approach. The training and validation datasets were used to develop the CNN model. The testing dataset comprised an independent set of images from patients whose images were not used for the development of the model. Figure 1 shows a flowchart summarizing the study design (Figure 2). The probability that the trained CNN would attribute each of the three categories to an image (excellent, satisfactory or unsatisfactory) was calculated.

The main outcome measures encompassed sensitivity and specificity to distinguish the three cleanliness categories, along with the positive predictive value (PPV), negative predictive value (NPV) and overall accuracy. Additionally, we assessed the model’s performance using the area under the receiver operating curve (AUROC) to gauge its ability to differentiate these categories. To account for potential data imbalances in our dataset, we examined the precision–recall (PR) curves and calculated the area under the PR curve (AUPRC) as a metric for the model’s discriminative capability. The CNN’s classifications were compared to those established by expert consensus, which is considered the gold standard. Furthermore, we evaluated the network’s image processing efficiency by measuring the time it took for the CNN to classify all validation images in the dataset. All statistical analyses were conducted using Sci-Kit Learn version 22.2 software. [14].

## 3. Results

### 3.1. Construction of the CNN

**Figure 3:**
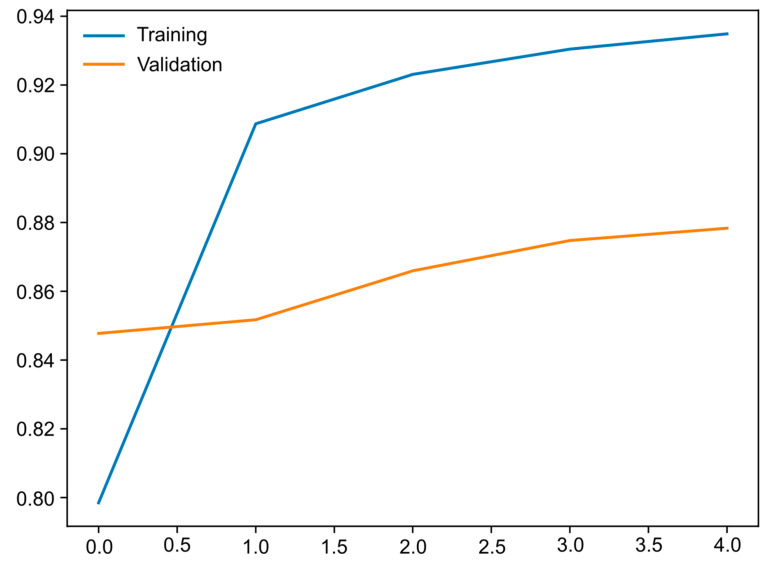
Evolution of the accuracy of the convolutional neural network along the epochs during training and validation phases, as the training and validation datasets were repeatedly inputted in the neural network.

In total, 35,269 colonic images were extracted for analysis from 141 CE examinations performed at the two clinical centers. Of the 141 procedures undertaken, 76 were carried out using the Pillcam Crohn’s Capsule™ (*n* = 17,297 images), 43 were carried out with the Pillcam SB3™ (*n* = 9442 images), 17 were carried out with the OMOM HD capsule™ (*n* = 8100 images) and 5 were carried out with the Pillcam COLON2™ system (*n* = 430 images). From this cohort of examinations, 13,382 were labeled by the experts as unsatisfactory preparation, 12,652 were labeled as satisfactory preparation and 9235 were labeled as excellent preparation. The training and validation datasets were built for the design of the CNN, incorporating 70% (*n* = 26,527) and 20% (*n* = 6725). The CNN was used to evaluate each image and attribute a predicted classification to it (excellent, satisfactory or unsatisfactory), which was then compared with that attributed to the image by the experts. As the data were repeatedly used as inputs to the multilayer CNN, the overall accuracy of the network was not only enhanced in the training period but also in the validation environments (Figure 3), reflecting the ability of the CNN to learn.

### 3.2. Global Performance of the CNN to Differentiate the Classes of Colonic Preparation

The CNN’s performance was evaluated using an independent dataset of images (Table 1). In summary, the deep learning algorithm demonstrated its ability to automatically distinguish between classes of colon preparation with an overall accuracy of 95.0% (95% CI, 91.4–98.5), a sensitivity of 91.4% (95% CI, 79.9–100.0), a specificity of 96.8% (95% CI, 93.8–99.8), a positive predictive value (PPV) of 93.7% (95% CI, 90.2–97.1) and a negative predictive value (NPV) of 96.1% (95% CI 92.0–100.0).

**Figure 4:**
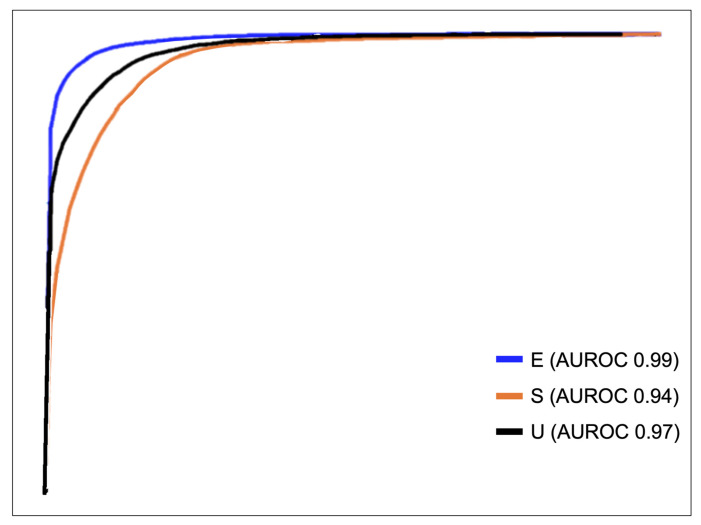
Receiver operating characteristic (ROC) curve of the convolutional neural network’s performance in differentiating the colonic preparation classes: AUROC, area under the ROC curve.

**Figure 5:**
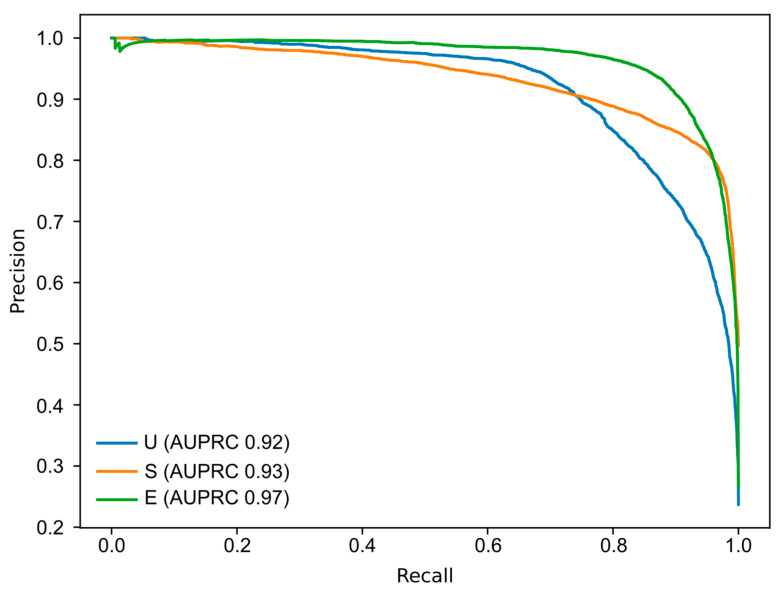
Precision–recall curves for the assessment of the discriminating capacity of the convolutional neural network for each colonic preparation class. AUPRC, area under the precision–recall curve.

The AUROCs underlined the CNN’s excellent performance in distinguishing between the different levels of colon cleanliness. Specifically, the AUROCs for excellent, satisfactory and unsatisfactory colon cleanliness were notably high, measuring 0.99, 0.94 and 0.97, respectively (Figure 4). These results were concordant with the analysis of the PR curves, which showed AUPRCs of 0.97, 0.93 and 0.92, respectively (Figure 5).

### 3.3. Computational Performance of the CNN

The CNN processed the testing dataset in 3 s, equating to a reading rate of 672 frames per second. By extrapolating this level of performance to a comprehensive CCE examination comprising 50,000 frames, it would take an estimated 74 s to complete the entire analysis.

## 4. Discussion

It is thought that the introduction of tools driven by AI will potentially revolutionize medical imaging, and CCE is well suited to such novel developments. In this study, we demonstrate the capacity of a multi-layered CNN-based AI tool to accurately and automatically categorize colonic cleanliness in CE examinations, with high levels of sensitivity and specificity. We believe that the tool represented in this study may significantly enhance the yield and efficacy of CCE procedures, the implementation of which, in regular clinical practice, will represent an important milestone for a widespread adoption of CE for a primary diagnostic exploration of the colon.

In order to successfully detect abnormalities in CCE examinations and ensure that they are conclusive, it is essential to achieve adequate bowel preparation [12,15,16,17]. Despite the importance of adequate colon preparation and the availability of different cleanliness grading scales with distinct technical characteristics [18,19], there is still no consensus for an objective and reliable scoring system to assess colon cleanliness following CCE or colonoscopy preparation. At present, the Boston Bowel Preparation Scale (BBPS) is considered the best validated scale for colon cleansing when assessed via a colonoscopy [20], although, like any subjective classification, it is highly observer-dependent. For the validation of this technology, we adopted a three-tier scale based on the proportion of the image in which the colon mucosa was visible as an attempt to achieve a more objective measure. Importantly, there is still no agreement on the most appropriate protocol for the preparation of colon examinations, an issue that is particularly important for CCE, given the impossibility of performing the washing and removal of debris during CCE procedures [21,22,23]. Hence, a pivotal component in the endeavor to automate the assessment of CE examinations will be the development of a system to assess the gastrointestinal tract’s cleanliness based on extracted images. In fact, in the future, the significance of colon cleanliness will continue to grow, as it plays a crucial role in ensuring that AI applications designed to evaluate the colon mucosa using various deep learning models can consistently deliver exceptional diagnostic outcomes. The CNN model developed in this study was trained using a large dataset of 35,269 real-world images obtained at two large-volume centers in order to enhance the variability of the dataset and, thus, its representativity. Importantly, we included images from four different CE devices, thus making this algorithm the first multibrand model designed for the assessment of colonic preparation during CE examinations. This constitutes an important step, particularly considering the growing interest in one-step minimally invasive capsule panendoscopy. All of the images were reviewed by CE experts, and the inclusion of each frame was dependent on the agreement of at least two of the experts. In fact, the greater the quantity and diversity of images employed for algorithm training, the more effective it becomes, mirroring the diverse scenarios encountered in real clinical practice. In addition, varied datasets are less likely to introduce bias into the performances of these tools, which is a phenomenon of considerable concern when considering the clinical implementation of such tools [24,25]. The performance of the CNN was assessed using an independent dataset of 2017 images, demonstrating an overall good performance in differentiating different levels of colon preparation based on a simple three-level classification scale of cleanliness defined through the relative proportion of the mucosa that can be visualized in each image. Testing the CNN revealed it to be accurate (95.0%), sensitive (91.4%) and specific (96.8%) relative to the gold standard. In addition, the CNN showed a good discriminating performance with AUROCs between 0.94 and 0.99 and AUPRCs between 0.92 and 0.97.

The CNN developed here performed similarly to the recent applications exploring CNN architectures to automatically assess cleanliness in CE examinations, such as when classifying images into four categories of cleanliness based on intestinal content (Noorda et al. 2020 [26]). That CNN model was trained on a large number of images (*n* = 55,293) but was tested on fewer images (*n* = 854) from 30 examinations performed in a clinical setting. Our group developed a similar algorithm for the classification of bowel preparation for small-bowel CE examinations. Indeed, our algorithm classified bowel preparation according to the same classification system and achieved a sensitivity of 88%, a specificity of 92% and an overall accuracy of 89%. Another neural-network-based algorithm trained on only 600 small-bowel images used a 10-point scale to categorize cleanliness as adequate or inadequate [27]. This algorithm had apparently worse sensitivity (90.3%), specificity (83.3%) and accuracy (89.7%), although it was based on the assessment of 156 CE recordings. Another deep learning algorithm that was developed to evaluate the clarity of the SB mucosa visualized according to a five-point scoring system, trained on a large sample of images (71,191) and verified on 120,000 images, proved to be less accurate [28,29].

Although there is a limited number of studies dedicated to automatically evaluate the cleanliness in CCE preparations through deep learning applications, this study presents certain noteworthy aspects and limitations. Firstly, it is important to highlight that this CNN was applied to four different CE systems, each with specific optical specifications and performance levels. While this demonstrates the versatility of the CNN across various systems, this study did not assess its performance on each system separately. Such an analysis could reveal whether there are variations in its performance when applied to different CCE systems, potentially influencing the model’s generalizability. Furthermore, the utilization of images captured at two different centers provides some evidence that the algorithm may be replicable in diverse clinical contexts. However, it is essential to conduct further studies to confirm this hypothesis. One significant limitation is the retrospective nature of this study, as it involves a relatively modest sample of patients. To establish the validity and reproducibility of our tool in real-world clinical practice, larger-scale, multicenter, prospective studies are required. Additionally, as the CNN was developed using still frames, it is imperative to evaluate its performance with full-length videos before integrating it into CCE examinations in clinical practice.

Capsule endoscopy has evolved into a valid alternative for colon examinations. However, despite recent technological advancements, this method still imposes time constraints and demands significant effort from the reading gastroenterologist. This labor-intensive and repetitive task is characterized by a limited reproducibility, potentially leading to the oversight of small lesions or abnormalities that may only appear in a few frames. Deep learning methods, such as CNNs, offer genuinely cost-effective solutions that can free up valuable resources, enhance diagnostic accuracy and yield improved results [30,31]. Ideally, AI algorithms designed for the automatic classification of bowel preparation should be seamlessly integrated into CCE interpretation alongside AI algorithms that can differentiate normal and abnormal mucosal images. This integration allows for the automatic filtering of images with normal mucosa and those with inadequate cleanliness, allowing gastroenterologists to concentrate on suspected lesions. As a result, the diagnostic efficacy is enhanced, the workload on gastroenterologists in terms of time and effort is lightened and the associated costs are reduced.

## 5. Conclusions

The adoption of minimally invasive CCE has represented an important advancement in clinical colon endoscopy, and the next major step forward is likely to involve the incorporation of AI tools into CCE protocols to automate the reading of these exams. A CNN-based model was developed to automatically classify colon preparation for CCE examinations based on a simple scale. The implementation of such automated systems to assess colon cleanliness in CCE should provide an optimization of the reading process of these exams, expanding the role of CE towards panendoscopy.

## Figures and Tables

**Table 1:** Confusion matrix of the automatic prediction of colon cleanliness versus expert classification.

		Expert Classification		
		Unsatisfactory	Satisfactory	Excellent
CNN classification	Unsatisfactory	948	96	0
	Satisfactory	27	392	10
	Excellent	0	37	507

Abbreviations: CNN—convolutional neural network.

## Data Availability

The data are available upon reasonable request.
